# Risk of homelessness after prison release and recidivism in Denmark: a nationwide, register-based cohort study

**DOI:** 10.1016/S2468-2667(23)00152-4

**Published:** 2023-08-25

**Authors:** Sandra Feodor Nilsson, Merete Nordentoft, Seena Fazel, Thomas Munk Laursen

**Affiliations:** Copenhagen Research Center for Mental Health (CORE), Mental Health Centre Copenhagen, Copenhagen University Hospital, Hellerup, Denmark; Copenhagen Research Center for Mental Health (CORE), Mental Health Centre Copenhagen, Copenhagen University Hospital, Hellerup, Denmark; Department of Clinical Medicine, University of Copenhagen, Copenhagen, Denmark; The Lundbeck Foundation Initiative for Integrated Psychiatric Research (iPSYCH), Aarhus, Denmark; Department of Psychiatry, University of Oxford, Oxford, UK; Oxford Health NHS Foundation Trust, Warneford Hospital, Oxford, UK; The National Centre for Register-Based Research, Aarhus University, Aarhus, Denmark

## Abstract

**Background:**

Transitional periods between and across services have been linked to homelessness. We aimed to investigate the association of previous history of homelessness and psychiatric disorders with risk of homelessness after release from prison. Additionally, we examined the association between homelessness after release and risk of recidivism.

**Methods:**

We did a nationwide, register-based cohort study of people aged 15 years or older who were released from prison for the first time in Denmark between Jan 1, 2001, and Dec 31, 2021. We obtained data using the Danish Civil Registration System with data linked across other registries (the Danish Central Criminal Register, the Danish Homeless Register, the Danish National Patient Register, and the Danish Psychiatric Central Research Register) on release date, homeless shelter contacts, psychiatric disorders, and new convictions. Outcomes were homelessness after release from prison, defined as first homeless shelter contact following release from first imprisonment, and recidivism within 2 years of release, defined as the first police-recorded criminal conviction after prison release. We calculated incidence rates per 1000 person-years, incidence rate ratios (IRRs) using Poisson regression analysis, and probability of homelessness and recidivism after release. Sex, age, calendar year, country of origin, highest educational level, relationship status, and length of index imprisonment were included as confounders.

**Findings:**

The study cohort included 37 382 individuals (34 792 males [93·1%] and 2590 females [6·9%]) aged 15–41 years, who were released from prison between Jan 1, 2001, and Dec 31, 2021, contributing 202 197 person-years at risk. Mean follow-up duration was 5·4 person-years (SD 5·6). Overall, 1843 (4·9%) of 37 382 individuals became homeless. 1 year after release from prison, 788 (2·1%) of 37 382 individuals had at least one homeless shelter contact, and among 1761 individuals with previous history of homelessness before index imprisonment, 357 (20·7%) became homeless. The incidence of homelessness after release was 102·5 cases per 1000 person-years for individuals with previous history of homelessness and 6·7 cases per 1000 person-years in individuals without (IRR 16·4, 95% CI 14·8–18·2; adjusted for sex, age, and calendar year). Individuals who additionally had a mental illness had a higher risk of homelessness (IRR 22·6, 19·7–25·9) compared with those without either previous homelessness or mental illness, and a substantially higher risk was observed for those with previous homelessness and drug use disorder (25·0, 21·6–28·9) compared with those without. Within 2 years of release from prison, the probability of recidivism was 73·2% (95% CI 72·8–73·7). The risk of recidivism was higher among people experiencing homelessness after release from prison than those who did not experience homelessness after release (IRR 1·5, 95% CI 1·3–1·7), adjusted for sex, age, and calendar year.

**Interpretation:**

Criminal justice services should review approaches to reduce risk of homelessness, and consider improving liaison with mental health and substance misuse services to prevent adverse outcomes on release from prison. Clinical guidelines applied to criminal justice settings should address the health of individuals who experience homelessness.

**Funding:**

Lundbeck Foundation.

## Introduction

The number of individuals experiencing homelessness is increasing in high-income countries, particularly among young people.1 Homelessness is associated with markedly reduced life expectancy, and higher rates of psychiatric disorders and violent victimisation than in the general population.^1–4^ Transitional periods have been identified as high-risk periods for homelessness.^5^ The increased likelihood of homelessness is partly explained by institutional barriers to obtaining stable housing.^6,7^

People who are incarcerated represent another marginalised population, and the number of individuals in prison is increasing: at present, more than 11 million individuals are in custody worldwide.^8^ The risk of homelessness is four times higher in individuals with a history of incarceration than in the general population.^9^ Findings from a meta-analysis showed that almost a fifth of people who are incarcerated experienced homelessness at the time of imprisonment, and around 30% on release.^10^ The prevalence of psychiatric disorders is high among people in prison,^11^ which further increases the risk of homelessness.^5,9^ Psychiatric disorders are also associated with increased risk of multiple incarcerations and re-offending, especially among people with schizophrenia and bipolar disorder.^12,13^ Unstable housing further increases the risk of recidivism in people with severe mental illness^14^ and those on probation.^15^ However, it is unknown whether there is an increased risk of recidivism after release related to homelessness with and without a psychiatric diagnosis. Under-detected and untreated psychiatric morbidity is common among individuals in prison in high-income countries.^16,17^ The importance of community-based mental health services on release has been outlined, but remains poorly implemented and under-resourced in many countries.^17^ Most studies of homelessness are limited by selected study cohorts, short follow-up, and absence of adjustment for confounding.^7,9,14,15^ The use of Danish registers to study homelessness after release is a unique opportunity.^2^

To assess the possible associations between psychiatric disorders and homelessness in people released from prison, we aimed to investigate whether a history of homelessness and psychiatric disorders increased the risk of homelessness after release from prison, overall and by sex. We also aimed to examine the association between homelessness after release from prison and recidivism. We hypothesised that in the year after prison release, individuals would be at high risk for homelessness,^8^ and that psychiatric disorders would increase the risk of homelessness.^5,7,9,14^

## Methods

### Study design and participants

We did a nationwide, register-based cohort study, which included all Danish residents aged at least 15 years (ie, the age of criminal responsibility in Denmark^18^), being at risk at least 1 day after release, born between Jan 1, 1980 and Dec 31, 2007, who were serving a prison sentence or were on remand in a jail or prison with a release date between Jan 1, 2001, and Dec 31, 2021. Individuals who were arrested, but not convicted or remanded to prison were not included. Due to issues with data availability on homelessness, the study cohort represent a younger subcohort than the entire Danish prison population.

The study was approved by the Danish Data Protection Agency, and data access was agreed by Statistics Denmark and the Danish Health Data Authority. Ethical approval and written informed consent were not required, as per Danish regulations for register-based studies. All data were de-identified and pseudo-anonymised at the individual level.

### Data sources

Data were retrieved from the Danish Civil Registration System, which contains information on the entire Danish population.^19^ This nationwide population register contains individual-level information on date of birth, death, and emigration, sex, and country of origin. All Danish residents are assigned a personal identification number (CPR number) at birth or start of residency, which ensures optimal linkage at an individual level between the different Danish population-based registers,^19^ including the Danish Central Criminal Register, the Danish Homeless Register, the Danish National Patient Register, and the Danish Psychiatric Central Research Register (appendix p 1).

Homelessness before the index imprisonment was the main exposure, defined as any homeless shelter contact of a minimum of 1 day. Each stay in a homeless shelter under the Social Service Act Section 110 requires a valid CPR number and costs a minor fee per night.^20^ The Danish municipalities are obliged to offer this type of accommodation to all individuals who have no home or cannot live in their home and are in need of extra support. The majority of people experiencing homelessness in Denmark have a valid CPR number and use the homeless shelters at some point, but undocumented migrants are likely to use night shelters or be street homeless. In a 2022 national survey, it was estimated that 5789 individuals were homeless in a given week in Denmark, corresponding to 0·1% of the general population. Around 47% of this population were using homeless shelters, 9% were street homeless, 4% were in night shelters, and 20% were staying with family and friends. An additional 322 individuals were estimated to be migrants.^21^

The definition of homelessness used in this study is broad and included different types of homelessness, but primarily was defined as being houseless (ie, people in accommodation for homelessness) as per the definition of homelessness used by the European Federation of National Organisations Working with the Homeless.^22^ People who were sleeping rough or sofa-surfing, who never visited the homeless shelters, were not included. The number of homeless shelter contacts before the index imprisonment was also studied.

Additionally, we studied individuals experiencing home-lessness who had psychiatric disorders (ie, an F diagnosis in the International Classification of Diseases [ICD], version 10 or equivalent ICD-8 diagnoses before 1994) before index imprisonment in the following groups: mental illness, severe mental illness (ie, schizophrenia spectrum disorder or bipolar disorder, alcohol use disorder, or drug use disorder), and comorbidity substance misuse (ie, mental illness and alcohol or drug use disorder; appendix pp 1–3).

### Outcomes

We studied two outcomes. The first outcome was homelessness after release from prison, defined as first homeless shelter contact following release from first imprisonment. The second outcome was recidivism within 2 years of release, defined as the first police-recorded criminal conviction after prison release. This analysis was furthermore stratified by type of conviction (criminal code violations, traffic violations, and other violations including weapon and illicit drug use violations).

### Statistical analysis

Individuals were at risk during the time period after first release from prison. We calculated the probability of becoming homeless during the first year after first prison release, overall and by sex and history of homelessness.

Additionally, we examined the probability of recidivism, within 2 years of release. Individuals were aged at least 15 years at the beginning of these analyses. We used the Aalen-Johansen estimator to estimate cumulative incidence functions considering competing risks from death and emigration. Second imprisonment was handled as a competing risk in the analysis of homelessness (ie, a person was only at risk during the period after first release). To test whether differences between groups in these analyses were statistically significant at a type I error rate of 5%, we used Gray’s test. Aalen-Johansen curves were smoothed to ensure no individuals could be identified by an event.

We also analysed data using a Poisson regression model with person-years at risk as an offset variable, thus approximating a Cox regression model.^23^ Basically and additionally adjusted incidence rate ratios (IRRs) were estimated from this model with Wald 95% CIs.^24^ First, we studied the association between previous homelessness independent of, and in combination, with psychiatric disorders according to the risk of homelessness after prison release. Cohort members were at risk from first prison release from Jan 1, 2001 and until an event occurred—ie, first homeless shelter contact, censoring due to emigration, death, second imprisonment, or study end (Dec 31, 2021).

Second, we studied the association between home-lessness after release independent of, and in combination with, psychiatric disorders and the incidence of recidivism. The cohort was followed up until outcome—ie, first date of any police-recorded conviction or censoring at emigration, death, 2 years of follow-up, or study end (Dec 31, 2021).

All analyses were adjusted for sex, age, and calendar year (model 1). A second model (model 2) was additionally adjusted for: country of origin, highest educational level (indicator of socioeconomic status), relationship status (ie, living with or without a partner 1 year before release; indicator of social network), and length of index imprisonment (indicator of severity of crime). For the analysis of homelessness before index imprisonment, any psychiatric disorder (including substance use disorder) was also included in model 2. The covariates were selected a priori because they were hypothesised to be confounders. Main results were stratified by sex.

We did four sensitivity analyses. First, we studied risk of homelessness without censoring at second imprison-ment to ascertain whether censoring had a substantial effect on the results. Second, we studied the risk of homelessness after release stratified by age groups (15–19, 20–29, and ≥30 years), and third, we stratified the analysis of homelessness after release by length of imprisonment (<7, 7–179, and ≥180 days). Fourth, we stratified the analyses of post-release homelessness and recidivism by remand status (serving a sentence or being on remand [ie, unsentenced individuals]).

All analyses were performed using SAS software (version 9.4).

### Role of the funding source

The funder of the study had no role in study design, data collection, data analysis, data interpretation, or writing of the manuscript.

## Results

Our study cohort included 37 382 individuals (34 792 males [93·1%] and 2590 females [6·9%]) released from prison in Denmark between Jan 1, 2001, and Dec 31, 2021, contributing 202 197 person-years at risk. Individuals were aged 15–41 years and the mean follow-up time was 5·4 person-years (SD 5·6). The median age of the cohort was 21·0 years (IQR 19·0–24·0). 1843 (4·9%) of 37 382 individuals experienced sheltered homelessness during the follow-up (appendix p 4); 1318 (71·5%) of 1843 individuals had no history of sheltered homelessness and 525 (28·5%) had a history of sheltered homelessness before index imprisonment.

The risk of sheltered homelessness was higher among people with a history of homelessness before the index imprisonment than in individuals from the general population with no previous shelter use (incidence 102·5 cases per 1000 person-years *vs* 6·7 cases per 1000 person-years; table).

**Figure 1:**
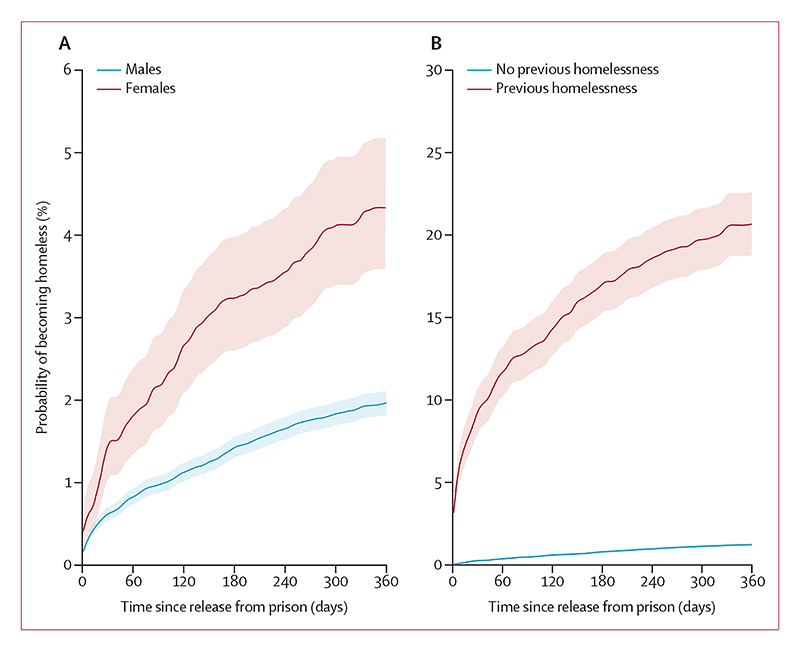
Probability of homelessness after first prison release, 2001–21 Probability of homelessness during one year after first prison release by sex (A) and by homelessness before index imprisonment (B). Competing risks from death, emigration, and second imprisonment were accounted for using the Aalen-Johansen estimator.

At 1-year follow-up, 788 (2·1%) of 37 382 individuals released from prison had at least one homeless shelter contact; 395 (1·1%) after only 90 days. The probability of becoming homeless after release was higher in females than males (110 [4·3%] of 2590 females *vs* 678 [2·0%] of 34 792 males after 1 year), and 357 (20·7%) of 1761 individuals with a history of homelessness before the index imprisonment became homeless within 1 year compared with 431 (1·2%) of 35 621 individuals with no previous history of homelessness(figure 1; appendix p 5).

**Figure 2:**
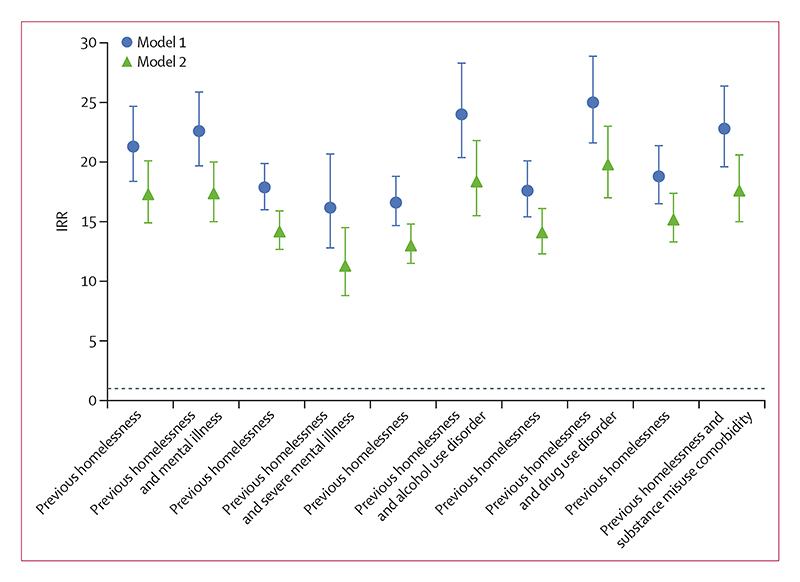
Homelessness after prison release, 2001–21 Risk of homelessness after first prison release by history of homelessness before index imprisonment, independent of, and in combination with, psychiatric disorders. Model 1 was adjusted for sex, age, and calendar time. Model 2 was additionally adjusted for country of origin, highest educational level, relationship status 1 year before release, and length of imprisonment. Error bars show 95% CIs. The dotted horizontal line shows the reference group (IRR=1) with no experiences of homelessness and without the individual psychiatric disorder examined. IRR=incidence rate ratio.

Homelessness before index imprisonment was also associated with a substantially increased risk of homelessness after release without considering psychiatric disorders, adjusted for sex, age, and calendar year (IRR 16·4 [95% CI 14·8–18·2]) compared with no previous homelessness. Individuals with homelessness before index imprisonment who also had an alcohol or a drug use disorder were at high risk for homelessness (IRR 24·0 [95% CI 20·4–28·3] for individuals with an alcohol use disorder; IRR 25·0 [95% CI 21·6–28·9] for individuals with a drug use disorder) compared with those without either (figure 2). The IRRs were slightly reduced after additional adjustment (model 2; appendix p 6).

The risks for homelessness after release by previous homelessness and psychiatric disorders were similar in males and females (appendix p 7).

The risk of homelessness after release was 38·7 times (95% CI 33·7–44·6) higher among individuals who had three or more homeless shelter contacts before index imprisonment than among individuals without a previous history of sheltered homelessness (appendix p 8). When assessing the risk of homelessness after release stratified by previous homelessness without censoring for second imprisonment, the risks were reduced, but remained higher than those for individuals without a previous history of homelessness (appendix p 9). Previous homelessness was associated with a higher risk of homelessness after release in individuals younger than 20 years than in the older age groups (IRR 31·6 [95% CI 20·8–48·1]; appendix p 10). The IRR of homelessness after release was higher in individuals who were incarcerated for less than 6 months than in those incarcerated for longer than 6 months (appendix p 11). When comparing risk of homelessness between people serving a sentence and people on remand, findings were overall similar to the main results: a history of homelessness increased risk of homelessness after release (appendix p 12).

**Figure 3:**
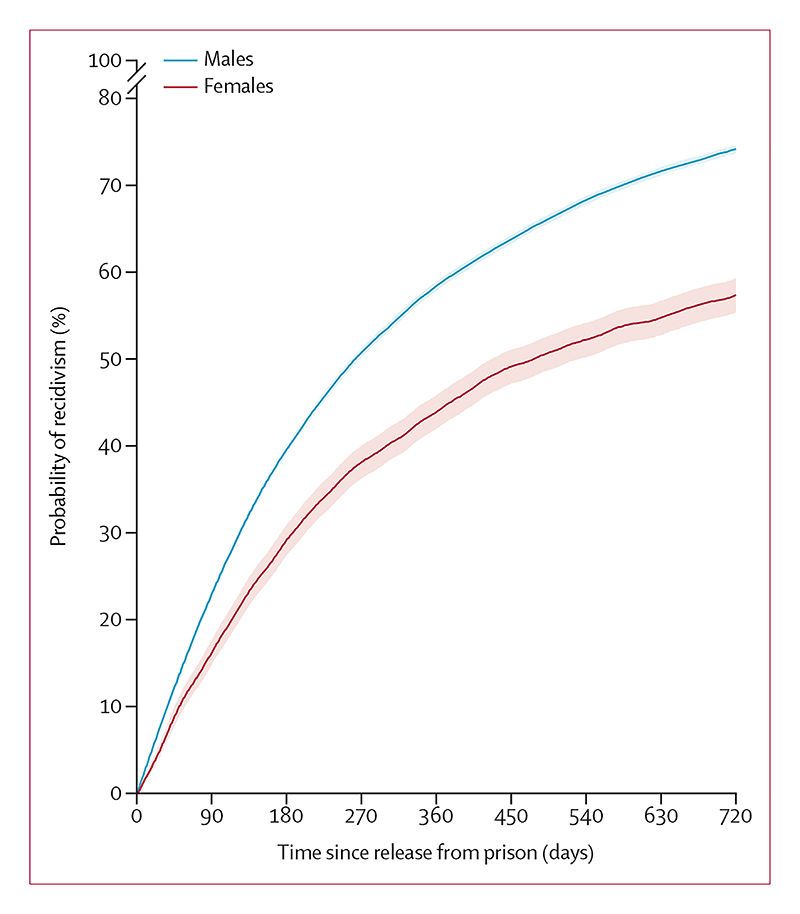
Probability of recidivism after first prison release, 2001–21 Probability of recidivism—ie, any police-recorded conviction, within 2 years of first prison release by sex. Competing risks from death and emigration were accounted for using the Aalen-Johansen estimator.

26 626 (73·2%; 95% CI 72·8–73·7) of 37 412 individuals released from their first imprisonment had any new police-recorded conviction within 2 years of release; cumulative incidence was higher in males (74·4%, 95% CI 73·9–74·8) than in females (57·7%, 95% CI 55·7–59·6; figure 3; appendix p 13). The incidence of recidivism after release was 166·8 cases per 1000 person-years.

**Figure 4:**
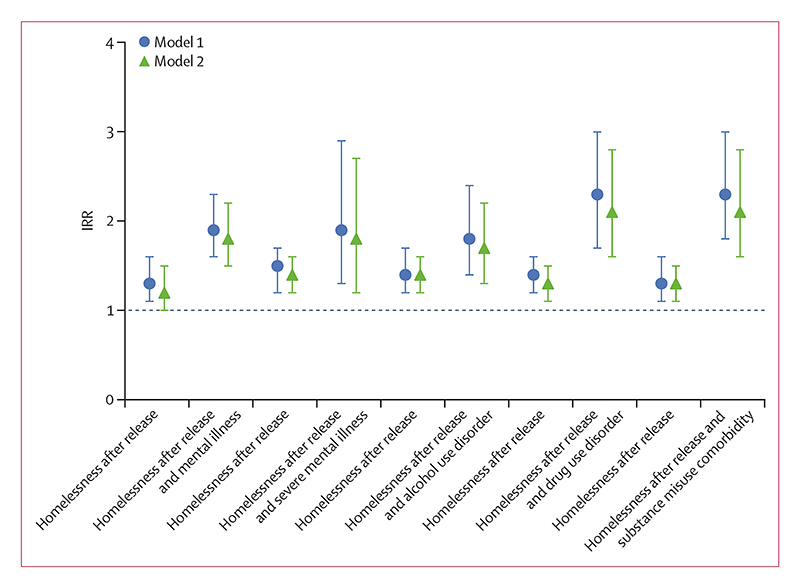
Recidivism after prison release, 2001–21 IRRs of recidivism within 2 years of first prison release by homelessness after release independent of, and in combination with, psychiatric disorders before index imprisonment. Model 1 was adjusted for sex, age, and calendar time. Model 2 was additionally adjusted for country of origin, highest educational level, relationship status 1 year before release, and length of index imprisonment. Error bars show 95% CIs. The dotted horizontal line shows the reference group (IRR=1) with no experiences of homelessness and without the individual psychiatric disorder examined. IRR=incidence rate ratio.

In model 1, the risk of recidivism was higher among people who were homeless after release than those who were not homeless after release (IRR 1·5 [95% CI 1·3–1·7]). The risk of recidivism was highest among individuals who experienced homelessness after release and who also had substance misuse comorbidity (IRR 2·3 [95% CI 1·8–3·0]) and drug use disorder (2·3 [1·7–3·0]) when compared with individuals without either (figure 4). Individuals experiencing homelessness after release who had a mental illness, severe mental illness, or alcohol use disorder had slightly lower, but increased risk of recidivism when compared with individuals with experiences of homelessness and mental illness, severe mental illness, or alcohol use disorder, respectively. Results remained almost unchanged after additional adjustment (model 2; appendix p 14). The highest risk of recidivism associated with homelessness after release was for criminal code violations (3·0, 95% CI 2·5–3·7; appendix p 15).

The risk for recidivism in females experiencing homelessness after release was 2·2 (95% CI 1·5–3·2) and in males 1·4 (95% CI 1·2–1·7). In absolute numbers, the risk was higher in males than females (226·0 cases per 1000 person-years among males experiencing homelessness *vs* 175·0 cases among males not experiencing homelessness; 198·6 cases among females experiencing homelessness *vs* 98·0 cases among females not experiencing homelessness). The risk of recidivism was highest among females experiencing homelessness who had an alcohol use disorder (4·5, 95% CI 2·4–8·2) when compared with females without either (appendix p 16).

Results for individuals serving a sentence were similar to the main results. For individuals on remand, the incidence of recidivism was higher among individuals with most of the psychiatric disorders in combination with homelessness and the highest risk of recidivism was observed among individuals with drug use disorders and homelessness (appendix p 17).

## Discussion

This register-based nationwide cohort study of 37 382 individuals released from prison between 2001 and 2021 in Denmark demonstrated that the period following release is a high-risk period for homelessness. People with a history of homelessness before imprisonment who also had a psychiatric disorder had a particularly high risk of homelessness. Around 2% of all individuals released from prison experienced homelessness within 1 year: 4% of females and 2% of males. Among people with a history of homelessness, there was a one in five chance of becoming homeless within the first year of release. We also investigated the association between experiencing homelessness after prison release and repeat offending, and found that homelessness was associated with a 1·5-times increased risk of recidivism, which was even higher among people with psychiatric morbidity. 73% of individuals released from prison re-offended within 2 years of release.

Our results are consistent with other studies done in high-income countries, which concluded that the period after prison release is a high-risk period for home-lessness,^7,9,25^ and many other adverse outcomes, including mortality,^26^ especially for individuals experiencing homelessness.^27^ Our findings are also consistent with previous studies of other transitional periods—eg, after discharge from psychiatric hospital.^5^ According to the findings of this study, although the prison population largely consists of males, women in prison constitute a population at high risk of becoming homeless.

From a broader public health perspective, these findings underscore the importance of accessible and effective mental health and substance misuse services targeted at people in prison with a history of homelessness, and more broadly in the community for people experiencing homelessness. Such interventions should include adequately resourced, multidisciplinary, and integrated mental health and substance misuse services. Release planning could consider homelessness risk in certain groups, linkage to community health services, and the involvement of social work and probation services in all individuals at high risk of becoming homeless. National guidelines for prison health should highlight the assessment and prevention of homelessness risk. Furthermore, preventing home-lessness remains a key public health priority and is associated with other outcomes, such as mortality, non-communicable diseases, and severe and enduring mental health conditions.^1^ We were not able to compare the absolute rates of homelessness with other countries because comparative data on the incidence of post-release homelessness were scarce.

Our results also confirmed that previous history of homelessness alone and in combination with psychiatric disorders, especially substance misuse, were strong predictors for homelessness after release from prison.^14,25,28^ These findings are consistent with previous evidence showing that substance use disorders are important predictors of homelessness in the general population,^9^ and in psychiatric inpatients after discharge.^5^ However, our results also indicated that individuals with previous homelessness and common mental illnesses are at high risk of homelessness after release from prison.

Although recidivism is difficult to compare between countries due to differences in definitions and study populations,^29^ the high probability of recidivism and the identification of homelessness as a predictor support previous findings.^14,15,30^ One study found that homelessness before imprisonment was a predictor of recidivism in people with problematic substance abuse^30^ and another study reported increased risk of recidivism in people experiencing homelessness during probation.^15^ In this study, 73% of individuals had at least one police-recorded crime within 2 years after release. We also demonstrated that females experiencing homelessness had a relatively higher risk of recidivism than males, especially those with psychiatric disorders. This confirms that females experiencing homelessness have multiple unmet needs with a high risk of adverse outcomes.^2,3^ Specifically for women leaving prison, efforts to prevent and mitigate the risk of intimate partner violence are important due to the additional risks for adverse outcomes, including poor mental health and suicide risk.^31^ Additionally, improving drug misuse treatment^32^ and considering the needs of the children of women leaving prison might be important areas to focus on since the prevention of the maltreatment of children might lead to a reduction in violence perpetration in the future.^13^ Homelessness and severe mental illness strongly predicted higher incidence of reincarceration.^14^ In this study, in a representative sample, we have shown that risk of recidivism is higher among people experiencing homelessness and psychiatric disorders. A Canadian study found that a housing-first intervention offered to adults experiencing homelessness with severe mental illness had no effect on the risk of incarceration.^33^ Little is known about release strategies that can effectively support people released from prison.

Our study had limitations. The use of register data might be associated with increased risk of misclassification of homelessness since we were only able to capture episodes of homelessness when people were using homeless shelters. People who did not use shelters but were experiencing homelessness were not counted as experiencing homelessness until they had a shelter contact, which is expected to lead to underestimation of the reported associations. Since we used the first homeless shelter contact as a measure of homelessness, we not only had individuals experiencing chronic homelessness, but also those more broadly who were socially marginalised.

We are not able to know if people experiencing homelessness after release without using shelters are less or more likely to re-offend, which could influence the association in both directions. Information on psychiatric disorders will be underestimated due to undiagnosed psychiatric disorders and due to lack of information from general practitioners (which is not included in the registers). This underestimation is expected to lead to conservative risk estimates for most psychiatric disorders. However, we are likely to have obtained comprehensive information on the most severe psychiatric disorders, since people would be expected to have accessed secondary services. Mental health can deteriorate during imprisonment, which was not captured.^34^ Technical violations of parole were included in the definition of recidivism, including all new crimes. Thus, there is a minor risk of misclassification of recidivism since these violations can occur for breaking parole conditions rather than committing a new offence. Residual confounding might exist. Information on treatment status, social network, childhood trauma, or individual coping strategy, housing availability, and community support was not included, because it is not reliably available in the registers used. Overestimation of effects might have occurred due to unmeasured confounding such as familial risks that predispose individuals to both criminal behaviour and homelessness. When studying associations in individuals on remand, the numbers of cases were relatively low in some subgroup analyses and could explain the absence of associations identified.

In Denmark, costs associated with outside housing are covered by municipalities within specific guidelines for the first 6 months that an individual is in prison.^35^ Strategies implemented between 2008 and 2010 aimed to improve the coordination of a released individual’s actions plan between the prison, the probation services, and the social services to address housing, employment, and treatment for alcohol and drug misuse.^18^ However, poor communication between municipalities and affordable housing providers has been reported.^36^ This might also explain why individuals with less than 6 months of imprisonment had a higher risk of homelessness than those with longer imprisonment. It is also likely that the risk of homelessness is underestimated in people with shorter imprisonments. However, this should be examined further. The involvement of other agencies to prevent homelessness is relevant, including substance misuse and mental health services, and ensuring their accessibility to marginalised and hard-to-reach populations needs review. Compared with other European countries, the estimated rate of incarceration in Denmark is low,^8,37^ however, it is likely that our results of the association between imprisonment and homelessness can be generalised to other European countries with social safety nets similar to the Danish welfare model because our study is representative of the Danish prison population aged 15–41 years. Our results cannot be generalised to people who do not have a Danish CPR number, which in 2021 was estimated to constitute around 11% of individuals in prison in Denmark.^36^ Studies from other countries are needed. We did not study the effect of health care on the risk of homelessness after release and recidivism. Homelessness and psychiatric disorders are expected to increase the risk of several other adverse outcomes after release from prison (eg, mortality) and is another area for future research.

In summary, this study indicates that there is a substantial need for improved prevention efforts addressing the risk of homelessness after prison release. Stable accommodation on release should be prioritised. Females released from prison constitute a particularly vulnerable group for homelessness. Psychiatric disorders contribute to increased risks of homelessness and recidivism after release. Thus, community services need to consider the combined risks of homelessness and the relapse of mental health conditions, which will be likely to require coordinated interventions across multiple agencies. Our results support the need for improved planning after release from prison to reduce social marginalisation.

## Supplementary Material

Appendix

## Figures and Tables

**Table:** Baseline sociodemographic characteristics

	No homelessness before index imprisonment				Homelessness before index imprisonment		
	Cases (n=1318)	Person-years (n=197 075), %	Incidence (95% CI)*		Cases (n=525)	Person-years (n=5122), %	Incidence (95% CI)*
Overall	··	··	6·7 (6·3–7·1)		··	··	102·5 (94·1·111·7)
Median age at time of release, years (IQR)	21·0 (19·0–24·0)	··	··		24·0 (21·0–28·0)	··	··
Age, years							
15–19	155 (11·8%)	15 514 (7·9%)	10·0 (8·5–11·7)		30 (5·7%)	72 (1·4%)	417·6 (292·0–597·3)
20–24	569 (43·2%)	54 971 (27·9%)	104 (9·5–11·2)		207 (39·4%)	1220 (23·8%)	169·6 (148·0–194·4)
25–29	319 (24·2%)	60 142 (30·5%)	5·3 (4·8–5·9)		154 (29·3%)	1698 (33·2%)	90·7 (77·5–106·2)
30–34	184 (14·0%)	43 855 (22·3%)	4·2 (3·6–4·9)		97 (18·5%)	1361 (26·6%)	71·3 (58·4–87·0)
≥35	91 (6·9%)	22 592 (11·5%)	4·0 (3·3–5·0)		37 (7·0%)	771 (15·1%)	48·0 (34·8–66·2)
Sex							
Male	1166 (88·5%)	181 379 (92·0%)	6·4 (6·1–6·8)		449 (85·5%)	4338 (84·7%)	103·5 (94·4–113·5)
Female	152 (11·5%)	15 697 (8·0%)	9·7 (8·3–11·4)		76 (14·5%)	784 (15·3%)	97·0 (77·4–121·4)
Country of origin^†^							
Denmark and other Western countries	1023 (77·6%)	152 693 (77·5%)	6·7 (6·3–7·1)		413 (78·7%)	4274 (83·4%)	96·6 (87·7–106·4)
Non-Western countries	295 (22·4%)	44 382 (22·5%)	6·6 (5·9–7·5)		112 (21·3%)	848 (16·6%)	132·1 (109·8–159·0)
Time period							
2001–05	113 (8·6%)	12 167 (6·2%)	9·3 (7·7–11·2)		50 (9·5%)	241 (4·7%)	207·8 (157·5–274·2)
2006–10	263 (20·0%)	37 114 (18·8%)	7·1 (6·3–8·0)		102 (19·4%)	771 (15·1%)	132·3 (109·0–160·6)
2011–15	339 (25·7%)	59 467 (30·2%)	5·7 (5·1–6·3)		143 (27·2%)	1474 (28·8%)	97·0 (82·4–114·3)
2016–21	603 (45·8%)	88 327 (44·8%)	6·8 (6·3–7·4)		230 (43·8%)	2637 (51·5%)	87·2 (76·7–99·3)
Relationship status 1 year before release							
Had a partner	391 (29·7%)	84 647 (43·0%)	4·6 (4·2–5·1)		93 (17·7%)	1188 (23·2%)	78·3 (63·9–95·9)
Single	180 (13·7%)	8146 (4·1%)	22·1 (19·1–25·6)		131 (25·0%)	650 (12·7%)	201·7 (170·0–239·4)
Single with children	747 (56^7%)	104 282 (52·9%)	7·2 (6·7–7·7)		301 (57·3%)	3284 (64·1%)	91·7 (81·9–102·6)
Highest educational level							
Higher education	176 (13·4%)	32 730 (16·6%)	5·4 (4·6–6·2)		41 (7·8%)	388 (7·6%)	105·8 (77·9–143·7)
Intermediate (high school or vocational training)	481 (36·5%)	87 085 (44·2%)	5·5 (5·1–6·2)		148 (28·2%)	1370 (26·7%)	108·0 (91·9–126·9)
Primary school or lower	661 (50·2%)	77 260 (39·2%)	8·6 (7·9–9·2)		336 (64·0%)	3364 (65·7%)	99·9 (89·8–111·2)
Psychiatric disorders before index imprisonment							
Any psychiatric disorder							
Yes	664 (50·4%)	53 597 (27·2%)	12·4 (11·5–13·4)		375 (71·4%)	3376 (65·9%)	111·1 (100·4–122·9)
No	654 (49·6%)	143 478 (72·8%)	4·6 (4·2–4·9)		150 (28·6%)	1746 (34·1%)	85·9 (73·2–100·8)
Any mental illness							
Yes	528 (40·1%)	37 949 (19·3%)	13·9 (12·8–15·2)		291 (55·4%)	2788 (54·4%)	104·4 (93·0–117·1)
No	790 (59·9%)	159 127 (80·7%)	5·0 (4·6–5·3)		234 (44·6%)	2334 (45·6%)	100·3 (88·2–114·0)
Any severe mental illness							
Yes	115 (8·7%)	5033 (2·6%)	22·8 (19·0–27·4)		71 (13·5%)	785 (15·3%)	90·5 (71·7–114·2)
No	1203 (91·3%)	192 042 (97·4%)	6·3 (5·9–6·6)		454 (86·5%)	4337 (84·7%)	104·7 (95·5–114·8)
Any alcohol use disorder							
Yes	247 (18·7%)	19 638 (10^0%)	12·6 (11·1–14·3)		170 (32·4%)	1391 (27·2%)	122·3 (105·2–142·1)
No	1071 (81·3%)	177 437 (90·0%)	6·0 (5·7–6·4)		355 (67·6%)	3731 (72·8%)	95·1 (85·7–105·6)
Any drug use disorder							
Yes	320 (24·3%)	14 807 (7·5%)	21·6 (19·4–24·1)		236 (45·0%)	1904 (37·2%)	124·0 (109·1–140·8)
No	998 (75·7%)	182 268 (92·5%)	5·5 (5·2–5·8)		289 (55·0%)	3218 (62·8%)	89·8 (80·0–100·8)
Any substance misuse comorbidity							
Yes	314 (23·8%)	14 663 (7·4%)	21·4 (19·2–23·9)		217 (41·3%)	1982 (38·7%)	109·5 (95·9–125·1)
No	1004 (76·2%)	182 413 (92·6%)	5·5 (5·2–5·9)		308 (58·7%)	3140 (61·3%)	98·1 (87·7–109·7)
Number of homeless shelter contacts before index imprisonment							
≥3	··	··	··		237 (45·1%)	1014 (19·8%)	233·8 (205·8–265·5)
2	··	··	··		93 (17·7%)	854 (16·7%)	108·8 (88·8–133·4)
1	··	··	··		195 (37·1%)	3254 (63·5%)	59·9 (52·1–69·0)
0	1318 (100·0%)	197 075 (100·0%)	6·7 (6·3–7·1)		··	··	··
Length of index imprisonment, months							
<1	690 (52·4%)	106 378 (54·0%)	6·5 (6·0–7·0)		254 (48·4%)	2538 (49·6%)	100·1 (88·5–113·2)
1 to <6	542 (41·1%)	81 383 (41·3%)	6·7 (6·1–7·2)		240 (45·7%)	2154 (42·1%)	111·4 (98·2–126·4)
≥6	86 (6·5%)	9315 (4·7%)	9·2 (7·5–11·4)		31 (5·9%)	429 (8·4%)	72·2 (50·8–102·7)
Type of index imprisonment							
Serving time	981 (74·4%)	152 708 (77·5%)	6·4 (6·0–6·8)		411 (78·3%)	4052 (79·1%)	101·4 (92·1–111·7)
On remand	337 (25·6%)	44 367 (22·5%)	7·6 (6·8–8·5)		114 (21·7%)	1070 (20·9%)	106·6 (88·7–128·1)
Type of index crime							
Sexual violent offence	32 (2·4%)	5315 (2·7%)	6·0 (4·3–8·5)		9 (1·7%)	129 (2·5%)	70·0 (36·4–134·5)
Violent offence	442 (33·5%)	84 260 (42·8%)	5·2(4·8–5·8)		150 (28·6%)	1596 (31·2%)	94·0 (80·1–110·3)
Property offence	551 (41·8%)	60 408 (30·7%)	9·1 (84·99		223 (42·5%)	1993 (38·9%)	111·9 (98·1–127·6)
Any other offence	293 (22·2%)	47 092 (23·9%)	6·2 (5·6–7·0)		143 (27·2%)	1404 (27·4%)	101·8 (86·4–120·0)

Due to rounding, the sum of person-years for each covariate might differ from the total number of person-years. *Per 1000 person-years. †Western countries including Andorra, Australia, Belgium, Bulgaria, Canada, Cyprus, Denmark, Estonia, Finland, France, Greece, Ireland, Iceland, Italy, Croatia, Latvia, Liechtenstein, Lithuania, Luxembourg, Malta, Monaco, the Netherlands, New Zealand, Northern Ireland, Norway, Poland, Portugal, Romania, San Marino, Switzerland, Slovakia, Slovenia, Spain, the UK, Sweden, Czech Republic, Germany, Hungary, the USA, Vatican City, and Austria. Non-Western countries include all other countries than those listed.

## Data Availability

The data that support the findings of this study are available from Statistics Denmark. The data access requires the completion of a detailed application form from the Danish Data Protection Agency, the Danish National Board of Health, and Statistics Denmark.

## Research in context

### Evidence before this study

We searched PubMed for studies of psychiatric disorders and homelessness published from database inception to May 2, 2022, using the mesh terms: “Prisons” and “Homeless Persons” OR “Homeless Youth”. We supplemented the search using the terms “incarcerat*” OR “prison*” OR “jail*” AND “homeless*” and scanned the reference lists of relevant articles. A large meta-analysis of predictors of homelessness included 19 studies of the association between imprisonment and risk of becoming homeless between 1988 and 2015 and reported a pooled odds ratio of 3·6 (95% CI 1·3–10·4) for homelessness risk. Other primary studies have found increased risk of housing instability in people who have left prison in the previous year and increased risk of recidivism associated with homelessness during probation. Psychiatric disorders have been associated with homelessness. However, links between homelessness, psychiatric disorders, and recidivism are not known, and few longitudinal studies have been done.

### Added value of this study

To the best of our knowledge, this is the first population-based study of the risk of homelessness after release from prison, and of the contribution of homelessness to repeat offending. We present sex-specific estimates and absolute and relative risks of homelessness after release from prison. We found that the period after prison release was an important period during which both male and female individuals were at high risk for homelessness. Female individuals and those with a history of homelessness were found to be at high risk for homelessness on release. Mental illness and substance misuse were identified as important predictors of homelessness in individuals released from prison, and further increased risk of recidivism in individuals experiencing homelessness.

### Implications of all the available evidence

Homelessness is an important adverse outcome to prevent after prison release, especially in individuals with psychiatric disorders, and in women. The link between homelessness and recidivism identified in our study suggests that the provision of supported housing after release and addressing psychiatric comorbidities should be prioritised. Community services need to consider homelessness, mental health, and substance misuse together, which will require multi-agency coordinated care after release.
