# Supplementary material for: Risk of homelessness after prison release and recidivism in Denmark: a nationwide, register-based cohort study
Source: Lancet Public Health. Author manuscript; Available in PMC 2025 Sep 2. (PMC7618063; doi:10.1016/S2468-2667(23)00152-4)
Supplement: Appendix [file EMS208267-supplement-Appendix.docx]

Appendix

|  | **Definition/categorisation** | **Data source** | **Register period** | **Register information available** | **Health authority** |
| --- | --- | --- | --- | --- | --- |
| **Outcomes** |  |  |  |  |  |
| Homelessness | First homeless shelter contact following release from first imprisonment | The Danish Homeless Register^1^ | 1999-2021 | Dates for homeless shelter contacts under the Consolidation Act of Social Services, Section 110. Each stay requires a valid CPR-number. | Statistics Denmark |
| Recidivism | First police-recorded criminal conviction after prison release recorded.  Types of convictions (the criminal code, traffic violations, and others incl. e.g., weapon and illicit drugs violations). | The Danish Central Criminal Register^2^ | 1980-2021 (information on release dates since 1991) | Information on all police-recorded crimes incl. imprisonment, release dates, and type of crime. Data for individuals with a valid civil registration number only. | Statistics Denmark |
| **Main exposure** | |  |  |  |  |
| Homelessness prior to index-imprisonment | Any stay in a homeless shelter prior to index-imprisonment measured from first date with a homeless shelter record.  Number of homeless shelter contacts prior to the index-imprisonment (0, 1, 2, 3+ shelter contacts). | The Danish Homeless Register^1^ | 1999-2021 | Dates for homeless shelter contacts under the Consolidation Act of Social Services, Section 110. Each stay requires a valid CPR-number. | Statistics Denmark |
| **Other main covariates** | |  |  |  |  |
| Psychiatric disorder incl. substance use disorders prior to index-imprisonment | Any F-diagnosis in the 10^th^ revision of the International Classification of Diseases (ICD) prior to release from first imprisonment | The Danish National Patient Register^3^ | 1995-2021 (information on outpatient and emergency room contacts since 1995; information on private contacts since 1995) | Public and private contacts. Inpatient contacts, outpatient, and emergency room contacts. Primary and secondary diagnoses. Dates for diagnosis. | Statistics Denmark and The Danish Health Data Authority |
| Psychiatric disorder incl. substance use disorders prior to index-imprisonment | Equivalent diagnoses to F-diagnosis in the 10^th^ revision of the International Classification of Diseases (ICD) from ICD version 8 prior to index-imprisonment | The Danish Psychiatric Central Research Register^4^ | 1969-1994 | Public contacts. Inpatient contacts, outpatient, and emergency room contacts. Primary and secondary diagnoses. Dates for diagnosis. | The Danish Health Data Authority |
| Substance use disorders prior to index-imprisonment | First date treated for any substance abuse. | The Registry of Drug Abusers Undergoing Treatment (SIB)^5^ | 1996-2020 | Dates of  treatment. | The Danish Health Data Authority |
| Substance use disorders prior to index-imprisonment | First date treated with injectable heroin or methadone. | The National Register on Treatment with Heroin and Methadone (IHM) | 2010-2020 | Dates of  treatment. | The Danish Health Data Authority |
| **Model 1 adjusting factors** | |  |  |  |  |
| Age | Time-dependent one-year groups | The Danish Civil Registration System^6^ | 1968-2021 | Containing information on basic personal information on all who hold a Danish civil registration number. | Statistics Denmark |
| Sex | Categorised as male and female | The Danish Civil Registration System^6^ | 1968-2021 | Containing information on basic personal information on all who hold a Danish civil registration number. | Statistics Denmark |
| Calendar time | Time-dependent one-year groups from 2001-2021 | The Danish Civil Registration System^6^ | 1968-2021 | Containing information on basic personal information on all who hold a Danish civil registration number. | Statistics Denmark |
| **Model 2 adjusting factors** | |  |  |  |  |
| Country of origin | Denmark, Other Western countries, and Non-Western countries defined according to Statistics Denmark’s categorisation, which can be found at Statistics Denmark’s homepage.^7^  Western Countries include: Andorra, Australia, Belgium, Bulgaria, Canada, Cyprus, Denmark, Estonia, Finland, France, Greece, Ireland, Iceland, Italy, Croatia, Latvia, Liechtenstein, Lithuania, Luxembourg, Malta, Monaco, The Netherlands, New Zealand, Northern Ireland, Norway, Poland, Portugal, Romania, San Marino, Switzerland, Slovakia, Slovenia, Spain, United Kingdom, Sweden, The Czech Republic, Germany, Hungary, USA, The Vatican State, Austria.  Non-Western Countries include all other countries than those listed above. | The Danish Civil Registration System^6^ | 1968-2021 | Containing information on basic personal information on all who hold a Danish civil registration number. | Statistics Denmark |
| Highest educational level | Highest educational level of the cohort members’ highest educational level at the age of 25 or the highest parental educational level at the time of the cohort member’s 15^th^ year birthday (i.e., if the individual was below 25 years old or no educational was completed) categorised as; higher educational level, intermediate level, primary school or lower. | The Integrated Database for Labour Market Research (IDA)^8^ | 1980-2021 | Containing information on highest completed educational level. | Statistics Denmark |
| Relationship status | Relationship status the year prior to release categorised as: having a partner, single, or single with children. | The Danish Civil Registration System^6^ | 1968-2021 | Containing information on basic personal information on all who hold a Danish civil registration number. | Statistics Denmark |
| Length of index-imprisonment | Length of first imprisonment categorised as: <1 month, 1-6 months, and 6+ months. | The Danish Central Criminal Register^2^ | 1980-2021 (information on release data since 1991) | Information on all police-recorded crimes incl. imprisonment, release dates, and type of crime. Data for individuals with a valid civil registration number only. | Statistics Denmark |
| **Other covariates** |  |  |  |  |  |
| Type of index-crime | Type of crime defined as: sexual violent offense, violent offense, property offense, and other offense. | The Danish Central Criminal Register^2^ | 1980-2021 (information on release data since 1991) | Information on all police-recorded crimes incl. imprisonment, release dates, and type of crime. Data for individuals with a valid civil registration number only. | Statistics Denmark |
| **Supplementary table 1. Information on covariates used in the study.** | | | | | |

| **Disease group** | **ICD-10 (Diagnosis time frame)** | **ICD-8** |
| --- | --- | --- |
| **Any psychiatric disorder including substance use disorders** | F00-F99 | 290-315 |
| Any mental illness | F00-F99 excl. alcohol and drug use disorders as defined below | 290-315 excl. alcohol and drug use disorders as defined below |
| Alcohol use disorder | F10  E24.4,  E52, G31.2, G62.1, G72.1, I42.6,  K29.2, K70, K85.2, K86.0,  O35.4,  Y57.3,  Z50.2, Z71.4, Z72.1 | 261.00, 262.00  291.x9,  303.x9, 303.20, 303.28, 303.90  571.00, 571.01 |
| Drug use disorder | F11-F19 | 294.39, 304.xx |
| Severe mental illness (schizophrenia spectrum disorders or bipolar disorders) | F20-F29; F30-31 | 295.x9, 296.89, 297.x9, 298.29.298.99, 299.04, 299.05, 299.09, 301.83; 296.19, 296.39, 298.19 |
| Substance misuse comorbidity (any mental illness and alcohol or drug use disorder) | F00-F99 excl. alcohol and drug use disorders  **AND**  (F10  E24.4,  E52, G31.2, G62.1, G72.1, I42.6,  K29.2, K70, K85.2, K86.0,  O35.4,  Y57.3,  Z50.2, Z71.4, Z72.1  **OR/AND**  F11-F19) | 290-315 excl. alcohol and drug use disorders  **AND**  (261.00, 262.00  291.x9,  303.x9, 303.20, 303.28, 303.90  571.00, 571.01  **OR/AND**  294.39, 304.xx) |
| **Supplementary table 2. Diagnostic codes used to define psychiatric disorders according to ICD-10 and ICD-8** | | |

Supplementary **figure 1. Flow diagram**

**Outcome 1: Post-release homelessness after first prison release**

Study population=37,382 individuals

Experienced outcome (homelessness) (n=1843)

Emigrated (n=529)

Imprisoned (n=17,746)

Died (n=332)

**Sub-analysis without censoring at second imprisonment:**

Study population (n=37,412 individuals)

Experienced outcome (homelessness) (n=3659)

Emigrated (n=999)

Died (n=748)

**Outcome 2: Recidivism after prison release**

Study population (n=37,412 individuals)

Experienced outcome (recidivism) (n=26,626)

Emigrated (n=90)

Died (n=164)

|  | |  |  | **90 days** | | **180 days** | | **360 days** | |
| --- | --- | --- | --- | --- | --- | --- | --- | --- | --- |
|  | | **Cases** | **Persons** | **CIF** | **(95% CI)** | **CIF** | **(95% CI)** | **CIF** | **(95% CI)** |
| **Total** | | 788 | 37,382 | 1.1 | (1.0-1.2) | 1.6 | (1.4-1.7) | 2.1 | (2.0-2.3) |
| **Sex** | |  |  |  |  |  |  |  |  |
| Males | | 678 | 34,792 | 1.0 | (0.9-1.1) | 1.4 | (1.3-1.6) | 2.0 | (1.8-2.1) |
| Females | | 110 | 2590 | 2.2 | (1.7- 2.8) | 3.2 | (2.6-4.0) | 4.3 | (3.6-5.2) |
| **Prior homelessness** | |  |  |  |  |  |  |  |  |
| Yes | 357 | | 1761 | 13.0 | (11.5-14.7) | 17.1 | (15.3-18.9) | 20.7 | (18.8-22.7) |
| No | 431 | | 35,621 | 0.5 | (0.4-0.5) | 0.8 | (0.7-0.9) | 1.2 | (1.1-1.3) |
| CIF=cumulative incidence function. P-values for the difference between groups were <0.0001.  ^*^The corresponding figure has been smoothed by using interpol sm=10 in SAS, proc gplot. | | | | | | | | | |
| **Supplementary table 3. Cumulative probability of homelessness after release from prison, 2001-2021^*^** | | | | | | | | | |

|  | **Cases** | **Person-years** | **IR/1000** | **IRR^*^** | **(95% CI)** | **IRR**^†^ | **(95% CI)** |
| --- | --- | --- | --- | --- | --- | --- | --- |
| **Total** | 1843 | 202,197 | 9.1 |  |  |  |  |
| **Prior homelessness** |  |  |  |  |  |  |  |
| Yes | 525 | 5122 | 102.5 | 16.4 | (14.8-18.2) | 10.1^‡^ | (9.1-11.3) |
| No | 1318 | 197,075 | 6.7 | 1 |  | 1 |  |
| **Prior homelessness in combination with psychiatric disorders** | | |  |  |  |  |  |
| **Mental illness** |  |  |  |  |  |  |  |
| Prior homelessness and mental illness | 291 | 2788 | 104.4 | 22.6 | (19.7-25.9) | 17.4 | (15.0-20.0) |
| Prior homelessness and no mental illness | 234 | 2334 | 100.3 | 21.3 | (18.4-24.7) | 17.3 | (14.9-20.1) |
| No prior homelessness and mental illness | 528 | 37949 | 13.9 | 2.6 | (2.3-2.9) | 2.5 | (2.2-2.8) |
| None | 790 | 159,127 | 5.0 | 1 |  | 1 |  |
| **Severe mental illness^¶^** |  |  |  |  |  |  |  |
| Prior homelessness and severe mental illness | 71 | 785 | 90.5 | 16.2 | (12.8-20.7) | 11.3 | (8.8-14.5) |
| Prior homelessness and no severe mental illness | 454 | 4337 | 104.7 | 17.9 | (16.0-19.9) | 14.2 | (12.7-15.9) |
| No prior homelessness and severe mental illness | 115 | 5033 | 22.9 | 3.7 | (3.0-4.5) | 3.14 | (2.6-3.8) |
| None | 1203 | 192,042 | 6.3 | 1 |  | 1 |  |
| **Alcohol use disorder** |  |  |  |  |  |  |  |
| Prior homelessness and alcohol use disorder | 170 | 1391 | 122.3 | 24.0 | (20.4-28.3) | 18.4 | (15.5-21.8) |
| Prior homelessness and no alcohol use disorder | 355 | 3731 | 95.1 | 16.6 | (14.7-18.8) | 13.0 | (11.5-14.8) |
| No prior homelessness and alcohol use disorder | 247 | 19,638 | 12.6 | 2.2 | (1.9-2.6) | 2.1 | (1.8-2.4) |
| None | 1071 | 177,437 | 6.0 | 1 |  | 1 |  |
| **Drug use disorder** |  |  |  |  |  |  |  |
| Prior homelessness and drug use disorder | 236 | 1904 | 124.0 | 25.0 | (21.6-28.9) | 19.8 | (17.0-23.0) |
| Prior homelessness and no drug use disorder | 289 | 3218 | 89.8 | 17.6 | (15.4-20.1) | 14.1 | (12.3-16.1) |
| No prior homelessness and drug use disorder | 320 | 14,807 | 21.6 | 3.9 | (3.4-4.4) | 3.6 | (3.1-4.1) |
| None | 998 | 182,268 | 5.5 | 1 |  | 1 |  |
| **Drug misuse incl. treatment registers^§^** |  |  |  |  |  |  |  |
| Prior homelessness and drug use disorder | 321 | 2790 | 115.0 | 26.1 | (22.9-29.7) | 21.2 | (18.5-24.3) |
| Prior homelessness and no drug use disorder | 204 | 2332 | 87.5 | 18.7 | (16.0-21.8) | 14.9 | (12.7-17.4) |
| No prior homelessness and drug use disorder | 460 | 23,628 | 19.5 | 3.8 | (3.4-4.3) | 3.6 | (3.2-4.1) |
| None | 858 | 173,447 | 4.9 | 1 |  | 1 |  |
| **Substance misuse comorbidity^¶^** |  |  |  |  |  |  |  |
| Prior homelessness and substance misuse comorbidity | 217 | 1982 | 109.5 | 22.8 | (19.6-26.4) | 17.6 | (15.0-20.6) |
| Prior homelessness and no substance misuse comorbidity | 308 | 3140 | 98.1 | 18.8 | (16.5-21.4) | 15.2 | (13.3-17.4) |
| No prior homelessness and substance misuse comorbidity | 314 | 14663 | 21.4 | 3.9 | (3.4-4.4) | 3.5 | (3.1-4.0) |
| None | 1004 | 182,413 | 5.5 | 1 |  | 1 |  |
| IR=incidence rate, IRR=incidence rate ratio  ^*^Adjusted for sex, age, and calendar time. ^†^Further adjusted for country of origin, highest educational level, single status the year prior to prison release, and length of imprisonment. ^‡^Further adjusted for any psychiatric disorder. ^¶^Severe mental illness defined as any schizophrenia spectrum disorder or any bipolar disorder; Substance misuse comorbidity defined as any mental illness and any alcohol or drug use disorder. ^§^Information on drug use disorder was besides from information from the Danish Psychiatric Central Research Register and the Danish National Patient Register also based on records from the Registry of Drug Abusers Undergoing Treatment and the National Register on Treatment with Heroin and Methadone. | | | | | | | |
| **Supplementary table 4. Incidence rate ratio of homelessness after prison release by homelessness and psychiatric disorders, 2001-2021** | | | | | | | |

|  | **Cases** | **Person-years** | **IR/1000** | **IRR^*^** | **(95% CI)** |
| --- | --- | --- | --- | --- | --- |
| **Males** |  |  |  |  |  |
| **Total** | 1615 | 185,717 | 8.7 |  |  |
| **History of homeless shelter contact** |  |  |  |  |  |
| Yes | 449 | 4338 | 103.5 | 17.3 | (15.5-19.3) |
| No | 1166 | 181,379 | 6.4 | 1 |  |
| **Prior homelessness in combination with psychiatric disorders** | | |  |  |  |
| **Mental illness** |  |  |  |  |  |
| Prior homelessness and mental illness | 242 | 2303 | 105.1 | 23.0 | (19.9-26.7) |
| Prior homelessness and no mental illness | 207 | 2036 | 101.7 | 22.3 | (19.1-26.1) |
| No prior homelessness and mental illness | 443 | 32,211 | 13.8 | 2.7 | (2.4-3.0) |
| None | 723 | 149,167 | 4.8 | 1 |  |
| **Severe mental illness^†^** |  |  |  |  |  |
| Prior homelessness and severe mental illness | 58 | 641 | 90.5 | 17.0 | (13.0-22.1) |
| Prior homelessness and no severe mental illness | 391 | 3698 | 105.7 | 18.7 | (16.7-21.1) |
| No prior homelessness and severe mental illness | 95 | 4099 | 23.2 | 4.0 | (3.3-5.0) |
| None | 1071 | 177,280 | 6.0 | 1 |  |
| **Alcohol use disorder** |  |  |  |  |  |
| Prior homelessness and alcohol use disorder | 140 | 1102 | 127.1 | 26.3 | (22.0-31.4) |
| Prior homelessness and no alcohol use disorder | 309 | 3237 | 95.5 | 17.0 | (15.0-19.4) |
| No prior homelessness and alcohol use disorder | 201 | 17,344 | 11.6 | 2.2 | (1.9-2.5) |
| None | 965 | 164,035 | 5.9 | 1 |  |
| **Drug use disorder** |  |  |  |  |  |
| Prior homelessness and drug use disorder | 190 | 1551 | 122.5 | 25.0 | (21.3-29.3) |
| Prior homelessness and no drug use disorder | 259 | 2787 | 92.9 | 18.8 | (16.3-21.6) |
| No prior homelessness and drug use disorder | 265 | 12,408 | 21.4 | 4.0 | (3.5-4.6) |
| None | 901 | 168,971 | 5.3 | 1 |  |
| **Substance misuse comorbidity^†^** |  |  |  |  |  |
| Prior homelessness and substance misuse comorbidity | 173 | 1605 | 107.8 | 22.3 | (18.9-26.4) |
| Prior homelessness and no substance misuse comorbidity | 276 | 2733 | 101.0 | 19.9 | (17.4-22.8) |
| No prior homelessness and substance misuse comorbidity | 253 | 12,007 | 21.1 | 3.9 | (3.4-4.5) |
| None | 913 | 169,372 | 5.4 | 1 |  |
| **Females** |  |  |  |  |  |
| **Total** | 228 | 16,480 | 13.8 |  |  |
| **History of homeless shelter contact** |  |  |  |  |  |
| Yes | 76 | 784 | 97.0 | 12.1 | (9.1-16.1) |
| No | 152 | 15,697 | 9.7 | 1 |  |
| **Prior homelessness in combination with psychiatric disorders** | | | | | |
| **Mental illness** |  |  |  |  |  |
| Prior homelessness and mental illness | 49 | 486 | 100.9 | 18.7 | (12.8-27.3) |
| Prior homelessness and no mental illness | 27 | 298 | 90.5 | 16.0 | (10.2-25.1) |
| No prior homelessness and mental illness | 85 | 5737 | 14.8 | 2.2 | (1.6-3.1) |
| None | 67 | 9959 | 6.7 | 1 |  |
| **Severe mental illness^†^** |  |  |  |  |  |
| Prior homelessness and severe mental illness | 13 | 144 | 90.2 | 12.2 | (6.8-21.8) |
| Prior homelessness and no severe mental illness | 63 | 640 | 98.5 | 13.3 | (9.8-18.1) |
| No prior homelessness and severe mental illness | 20 | 934 | 21.4 | 2.3 | (1.5-3.7) |
| None | 132 | 14,763 | 8.9 | 1 |  |
| **Alcohol use disorder** |  |  |  |  |  |
| Prior homelessness and alcohol use disorder | 30 | 289 | 103.8 | 16.7 | (11.1-25.3) |
| Prior homelessness and no alcohol use disorder | 46 | 495 | 93.0 | 14.1 | (9.9-20.0) |
| No prior homelessness and alcohol use disorder | 46 | 2295 | 20.0 | 2.6 | (1.9-3.7) |
| None | 106 | 13,402 | 7.9 | 1 |  |
| **Drug use disorder** |  |  |  |  |  |
| Prior homelessness and drug use disorder | 46 | 353 | 130.3 | 22.4 | (15.6-32.1) |
| Prior homelessness and no drug use disorder | 30 | 431 | 69.6 | 11.3 | (7.5-17.1) |
| No prior homelessness and drug use disorder | 55 | 2400 | 22.9 | 3.1 | (2.2-4.3) |
| None | 97 | 13,297 | 7.3 | 1 |  |
| **Substance misuse comorbidity^†^** |  |  |  |  |  |
| Prior homelessness and substance misuse comorbidity | 44 | 376 | 117.0 | 22.4 | (15.4-32.6) |
| Prior homelessness and no substance misuse comorbidity | 32 | 408 | 78.5 | 12.9 | (8.6-19.4) |
| No prior homelessness and substance misuse comorbidity | 61 | 2656 | 23.0 | 3.4 | (2.4-4.7) |
| None | 91 | 13,041 | 7.0 | 1 |  |
| IR=incidence rate, IRR=incidence rate ratio  ^*^Adjusted for sex, age, and calendar time. ^†^Severe mental illness defined as any schizophrenia spectrum disorder or any bipolar disorder; Substance misuse comorbidity defined as any mental illness and any alcohol or drug use disorder. | | | | | |
| **Supplementary table 5. Incidence rate ratio of homelessness after prison release by sex, 2001-2021** | | | | | |

|  |  |  |  |  |  |
| --- | --- | --- | --- | --- | --- |
|  | **Cases** | **Person-years** | **IR/1000** | **IRR^*^** | **(95% CI)** |
| **Total** | 1843 | 202,197 | 9.1 |  |  |
| **Country of origin** |  |  |  |  |  |
| Non-Western countries | 407 | 45,230 | 9.0 | 0.92 | (0.8-1.03) |
| Denmark and other Western countries | 1436 | 156,967 | 9.1 | 1 |  |
| **Living status** |  |  |  |  |  |
| Single | 311 | 8795 | 35.4 | 6.5 | (5.4-7.8) |
| Single with children | 1048 | 107,566 | 9.7 | 1.8 | (1.6-2.0) |
| Having a partner | 484 | 85,836 | 5.6 | 1 |  |
| **Highest educational level** |  |  |  |  |  |
| Primary school or lower | 997 | 80,624 | 12.4 | 2.2 | (1.9-2.6) |
| Intermediate (high school/vocational training) | 629 | 88,455 | 7.1 | 1.2 | (1.0-1.4) |
| Higher education | 217 | 33,118 | 6.6 | 1 |  |
| **Length of imprisonment** |  |  |  |  |  |
| 6 months or longer | 117 | 9744 | 12.0 | 1.5 | (1.2-1.8) |
| 1-6 months | 782 | 83,537 | 9.4 | 1.1 | (1.0-1.2) |
| < 1 month | 944 | 108,916 | 8.7 | 1 |  |
| **Type of crime** |  |  |  |  |  |
| Sexual violent offense | 41 | 5443 | 7.5 | 0.9 | (0.6-1.2) |
| Violent offense | 592 | 85,855 | 6.9 | 0.7 | (0.7-0.8) |
| Property offense | 774 | 62,402 | 12.4 | 1.3 | (1.1-1.4) |
| Other offense | 436 | 48,497 | 9.0 | 1 |  |
| **Number of homeless shelter contacts** |  |  |  |  |  |
| 3+ | 237 | 1014 | 233.8 | 38.7 | (33.7-44.6) |
| 2 | 93 | 854 | 108.8 | 17.8 | (14.4-22.0) |
| 1 | 195 | 3254 | 59.9 | 9.5 | (8.2-11.1) |
| 0 | 1318 | 197,075 | 6.7 | 1 |  |
| IR=incidence rate, IRR=incidence rate ratio  ^*^Adjusted for sex, age, and calendar time. | | | | | |
| **Supplementary table 6. Incidence rate ratio of homelessness after prison release, 2001-2021** | | | | | |

|  | **Cases** | **Person-years** | **IR/1000** | **IRR^*^** | **(95% CI)** |
| --- | --- | --- | --- | --- | --- |
| **Total** | 3659 | 363,070 | 10.1 |  |  |
| **Prior homelessness** |  |  |  |  |  |
| Yes | 741 | 8399 | 88.2 | 11.2 | (10.3-12.2) |
| No | 2918 | 354,671 | 8.2 | 1 |  |
| IR=incidence rate, IRR=incidence rate ratio  ^*^Adjusted for sex, age, and calendar time. | | | | | |
| **Supplementary table 7. Incidence rate ratio of homelessness after prison release without censoring individuals at their second imprisonment, 2001-2021** | | | | | |

|  | **Cases** | **Person-years** | **IR/1000** | **IRR^*^** | **(95% CI)** |
| --- | --- | --- | --- | --- | --- |
| **Total** | 1843 | 202,197 | 9.1 |  |  |
| **Age 15-19 years** |  |  |  |  |  |
| **Prior homelessness** |  |  |  |  |  |
| Yes | 30 | 72 | 417.6 | 31.6 | (20.8-48.1) |
| No | 155 | 15,514 | 10.0 | 1 |  |
| **Age 20-29 years** | | |  |  |  |
| **Prior homelessness** |  |  |  |  |  |
| Yes | 361 | 2918 | 123.7 | 16.1 | (14.3-18.3) |
| No | 888 | 115,113 | 7.7 | 1 |  |
| **Age 30+** |  |  |  |  |  |
| **Prior homelessness** |  |  |  |  |  |
| Yes | 134 | 2132 | 62.8 | 14.9 | (12.1-18.4) |
| No | 275 | 66,448 | 4.1 | 1 |  |
| IR=incidence rate, IRR=incidence rate ratio  ^*^Adjusted for sex, age, and calendar time. | | | | | |
| **Supplementary table 8. Incidence rate ratio of sheltered homelessness after prison release by homelessness prior to index-imprisonment and age-groups, 2001-2021** | | | | | |

|  | **Cases** | **Person-years** | **IR/1000** | **IRR^*^** | **(95% CI)** |
| --- | --- | --- | --- | --- | --- |
| **Total** | 1843 | 202,197 | 9.1 |  |  |
| **<7 days** |  |  |  |  |  |
| **Prior homelessness** |  |  |  |  |  |
| Yes | 78 | 788 | 99.0 | 14.3 | (11.0-18.5) |
| No | 276 | 38,073 | 7.2 | 1 |  |
| **7-179 days** | | |  |  |  |
| **Prior homelessness** |  |  |  |  |  |
| Yes | 416 | 3905 | 106.5 | 17.8 | (15.8-20.0) |
| No | 956 | 149,688 | 6.4 | 1 |  |
| **180+ days** |  |  |  |  |  |
| **Prior homelessness** |  |  |  |  |  |
| Yes | 31 | 429 | 72.2 | 8.5 | (5.6-12.9) |
| No | 86 | 9315 | 9.2 | 1 |  |
| IR=incidence rate, IRR=incidence rate ratio  ^*^Adjusted for sex, age, and calendar time. ^†^ | | | | | |
| **Supplementary table 9. Incidence rate ratio of sheltered homelessness after prison release by homelessness prior to index-imprisonment and length of index-imprisonment, 2001-2021** | | | | | |

|  | **Cases** | **Person-years** | **IR/1000** | **IRR^*^** | **(95% CI)** |
| --- | --- | --- | --- | --- | --- |
| **People serving a sentence** |  |  |  |  |  |
| **Total** | 1392 | 156,760 | 8.9 |  |  |
| **History of homeless shelter contact** |  |  |  |  |  |
| Yes | 411 | 4052 | 101.4 | 16.6 | (14.7-18.6) |
| No | 981 | 152,708 | 6.4 | 1 |  |
| **Prior homelessness in combination with psychiatric disorders** |  |  |  |  |  |
| **Mental illness** | 215 | 1955 | 110.0 | 24.7 | (21.0-29.0) |
| Prior homelessness and mental illness | 196 | 2098 | 93.4 | 20.9 | (17.8-24.6) |
| Prior homelessness and no mental illness | 390 | 27,159 | 14.4 | 2.9 | (2.5-3.3) |
| No prior homelessness and mental illness | 591 | 125,549 | 4.7 | 1 |  |
| None |  |  |  |  |  |
| **Severe mental illness^†^** |  |  |  |  |  |
| Prior homelessness and severe mental illness | 38 | 306 | 124.0 | 20.3 | (14.6-28.2) |
| Prior homelessness and no severe mental illness | 373 | 3746 | 99.6 | 17.3 | (15.3-19.5) |
| No prior homelessness and severe mental illness | 61 | 2361 | 25.8 | 4.2 | (3.2-5.4) |
| None | 920 | 150,347 | 6.1 | 1 |  |
| **Alcohol use disorder** |  |  |  |  |  |
| Prior homelessness and alcohol use disorder | 138 | 1145 | 120.5 | 24.1 | (20.0-29.0) |
| Prior homelessness and no alcohol use disorder | 273 | 2908 | 93.9 | 16.6 | (14.4-19.1) |
| No prior homelessness and alcohol use disorder | 186 | 16230 | 11.5 | 2.1 | (1.8- 2.5) |
| None | 795 | 136,478 | 5.8 | 1 |  |
| **Drug use disorder** |  |  |  |  |  |
| Prior homelessness and drug use disorder | 175 | 1310 | 133.6 | 27.0 | (22.8-31.9) |
| Prior homelessness and no drug use disorder | 236 | 2743 | 86.1 | 17.4 | (15.0-20.1) |
| No prior homelessness and drug use disorder | 232 | 10,941 | 21.2 | 3.9 | (3.3-4.5) |
| None | 749 | 141,767 | 5.3 | 1 |  |
| **Substance misuse comorbidity^†^** |  |  |  |  |  |
| Prior homelessness and substance misuse comorbidity | 158 | 1389 | 113.8 | 24.0 | (20.1-28.6) |
| Prior homelessness and no substance misuse comorbidity | 253 | 2664 | 95.0 | 18.8 | (16.3-21.8) |
| No prior homelessness and substance misuse comorbidity | 231 | 10,621 | 21.7 | 4.0 | (3.5-4.7) |
| None | 750 | 142,087 | 5.3 | 1 |  |
| **People on remand** |  |  |  |  |  |
| **Total** | 451 | 45,437 | 9.9 |  |  |
| **History of homeless shelter contact** |  |  |  |  |  |
| Yes | 114 | 1070 | 106.6 | 15.7 | (12.6-19.5) |
| No | 337 | 44,367 | 7.6 | 1 |  |
| **Prior homelessness in combination with psychiatric disorders** | | |  |  |  |
| **Mental illness** |  |  |  |  |  |
| Prior homelessness and mental illness | 76 | 834 | 91.2 | 17.5 | (13.3-23.0) |
| Prior homelessness and no mental illness | 38 | 236 | 161.1 | 27.1 | (19.1-38.4) |
| No prior homelessness and mental illness | 138 | 10790 | 12.8 | 2.1 | (1.6-2.6) |
| None | 199 | 33577 | 5.9 | 1 |  |
| **Severe mental illness^†^** |  |  |  |  |  |
| Prior homelessness and severe mental illness | 33 | 478 | 69.0 | 12.7 | (8.8-18.4) |
| Prior homelessness and no severe mental illness | 81 | 591 | 137.0 | 21.4 | (16.7-27.5) |
| No prior homelessness and severe mental illness | 54 | 2672 | 20.2 | 3.2 | (2.4-4.4) |
| None | 283 | 41,696 | 6.8 | 1 |  |
| **Alcohol use disorder** |  |  |  |  |  |
| Prior homelessness and alcohol use disorder | 32 | 246 | 130.2 | 23.7 | (16.3-34.6) |
| Prior homelessness and no alcohol use disorder | 82 | 824 | 99.5 | 16.4 | (12.7-21.1) |
| No prior homelessness and alcohol use disorder | 61 | 3408 | 17.9 | 2.7 | (2.1-3.6) |
| None | 276 | 40,959 | 6.7 | 1 |  |
| **Drug use disorder** |  |  |  |  |  |
| Prior homelessness and drug use disorder | 61 | 594 | 102.7 | 20.2 | (15.1-27.0) |
| Prior homelessness and no drug use disorder | 53 | 476 | 111.4 | 19.4 | (14.4-26.2) |
| No prior homelessness and drug use disorder | 88 | 3866 | 22.8 | 3.8 | (3.0-4.9) |
| None | 249 | 40,501 | 6.1 | 1 |  |
| **Substance misuse comorbidity^†^** |  |  |  |  |  |
| Prior homelessness and substance misuse comorbidity | 59 | 593 | 99.5 | 19.5 | (14.5-26.2) |
| Prior homelessness and no substance misuse comorbidity | 55 | 477 | 115.4 | 19.2 | (14.3-25.8) |
| No prior homelessness and substance misuse comorbidity | 83 | 4042 | 20.5 | 3.4 | (2.6-4.3) |
| None | 254 | 40,326 | 6.3 | 1 |  |
| IR=incidence rate, IRR=incidence rate ratio  ^*^Adjusted for sex, age, and calendar time. ^†^Severe mental illness defined as any schizophrenia spectrum disorder or any bipolar disorder; Substance misuse comorbidity defined as any mental illness and any alcohol or drug use disorder. | | | | | |
| **Supplementary table 10. Incidence rate ratio of homelessness after prison release by type of index-imprisonment, 2001-2021** | | | | | |

|  |  |  | **1 year** | | **2 years** | |
| --- | --- | --- | --- | --- | --- | --- |
|  | **Cases** | **Persons** | **CIF** | **(95% CI)** | **CIF** | **(95% CI)** |
| **Total** | 26,626 | 37,412 | 57.7 | (57.2-58.2) | 73.2 | 72.8-73.7 |
| **Sex** |  |  |  |  |  |  |
| Males | 25,195 | 34,820 | 58.7 | (58.2- 59.2) | 74.4 | (73.9-74.8) |
| Females | 1431 | 2592 | 44.2 | (42.2-46.1) | 57.7 | (55.7-59.6) |
| CIF=cumulative incidence function  ^*^Recidivism defined as any conviction within two years after release from first imprisonment. | | | | | | |
| **Supplementary table 11. Cumulative probability of recidivism following release from first imprisonment, 2001-2021^*^** | | | | | | |

|  |  |  |  |  |  |  |  |
| --- | --- | --- | --- | --- | --- | --- | --- |
|  | **Cases** | **Person-years** | **IR/1000** | **IRR^*^** | **95% CI** | **IRR**^†^ | **95% CI** |
| **Total** | 5879 | 35,256 | 166.8 |  |  |  |  |
| **Homelessness after prison release in combination with psychiatric disorders** | | |  |  |  |  |  |
| **Mental illness** |  |  |  |  |  |  |  |
| Homelessness and any mental illness | 96 | 368 | 261.1 | 1.9 | (1.6-2.3) | 1.8 | (1.5-2.2) |
| Homelessness and no mental illness | 101 | 522 | 193.6 | 1.3 | (1.1-1.6) | 1.2 | (1.0-1.5) |
| No homelessness and any mental illness | 1223 | 6877 | 177.8 | 1.2 | (1.1-1.3) | 1.2 | (1.1-1.2) |
| None | 4459 | 27490 | 162.2 | 1 |  | 1 |  |
| **Severe mental illness**^‡^ |  |  |  |  |  |  |  |
| Homelessness and any severe mental illness | 25 | 99 | 253.5 | 1.9 | (1.3-2.9) | 1.8 | (1.2-2.7) |
| Homelessness and no severe mental illness | 172 | 791 | 217.5 | 1.5 | (1.2-1.7) | 1.4 | (1.2-1.6) |
| No homelessness and any severe mental illness | 179 | 1084 | 165.1 | 1.1 | (1.0-1.3) | 1.0 | (0.9-1.2) |
| None | 5503 | 33283 | 165.3 | 1 |  | 1 |  |
| **Alcohol use disorder** |  |  |  |  |  |  |  |
| Homelessness and alcohol use disorder | 49 | 200 | 244.5 | 1.8 | (1.4-2.4) | 1.7 | (1.3-2.2) |
| Homelessness and no alcohol use disorder | 148 | 689 | 214.8 | 1.4 | (1.2-1.7) | 1.4 | (1.2-1.6) |
| No homelessness and alcohol use disorder | 653 | 3807 | 171.5 | 1.2 | (1.1-1.3) | 1.2 | (1.1-1.3) |
| None | 5029 | 30560 | 164.6 | 1 |  | 1 |  |
| **Drug use disorder** |  |  |  |  |  |  |  |
| Homelessness and drug misuse | 54 | 175 | 307.8 | 2.3 | (1.7-3.0) | 2.1 | (1.6-2.8) |
| Homelessness and no drug misuse | 143 | 714 | 200.3 | 1.4 | (1.2-1.6) | 1.3 | (1.1-1.5) |
| No homelessness and any drug misuse | 508 | 2468 | 205.8 | 1.5 | (1.3-1.6) | 1.4 | (1.3-1.5) |
| None | 5174 | 31899 | 162.2 | 1 |  | 1 |  |
| **Drug misuse incl. treatment registers**^¶^ |  |  |  |  |  |  |  |
| Homelessness and drug misuse | 76 | 294 | 258.9 | 1.9 | (1.5-2.4) | 1.8 | (1.5-2.3) |
| Homelessness and no drug misuse | 121 | 596 | 203.1 | 1.4 | (1.2-1.7) | 1.3 | (1.1-1.6) |
| No homelessness and any drug misuse | 809 | 3924 | 206.2 | 1.5 | (1.3-1.6) | 1.4 | (1.3-1.5) |
| None | 4873 | 30443 | 160.1 | 1 |  | 1 |  |
| **Substance misuse comorbidity**^‡^ |  |  |  |  |  |  |  |
| Homelessness and substance misuse comorbidity | 58 | 191 | 303.8 | 2.3 | (1.8-3.0) | 2.1 | (1.6-2.8) |
| Homelessness and no substance misuse comorbidity | 139 | 698 | 199.0 | 1.3 | (1.1-1.6) | 1.3 | (1.1-1.5) |
| No homelessness and substance misuse comorbidity | 526 | 2874 | 183.0 | 1.3 | (1.2-1.4) | 1.3 | (1.2-1.4) |
| None | 5156 | 31493 | 163.7 | 1 |  | 1 |  |
| IR=incidence rate, IRR=incidence rate ratio  ^*^Adjusted for sex, age, and calendar time. ^†^Further adjusted for country of origin, highest educational level, single status the year prior to prison release, and length of imprisonment. ^‡^Severe mental illness defined as any schizophrenia spectrum disorder or any bipolar disorder; Substance misuse comorbidity defined as any mental illness and any alcohol or drug use disorder. ^¶^Information on drug use disorder was besides from information from the Danish Psychiatric Central Research Register and the Danish National Patient Register also based on records from the Registry of Drug Abusers Undergoing Treatment and the National Register on Treatment with Heroin and Methadone. | | | | | | | |
| **Supplementary table 12. Incidence rate ratio of recidivism within two years after prison release by homelessness and psychiatric disorders, 2001-2021** | | | | | | | |

|  |  |  |  |  |  |  |  |
| --- | --- | --- | --- | --- | --- | --- | --- |
|  | **Cases** | **Person-years** | **IR/1000** | **IRR^*^** | **95% CI** | **IRR**^†^ | **95% CI** |
| **Total** | 5879 | 35,256 | 166.8 |  |  |  |  |
| **Any conviction** |  |  |  |  |  |  |  |
| Post-release homelessness | 197 | 889 | 221.5 | 1.5 | (1.3-1.7) | 1.4 | (1.2-1.6) |
| No sheltered homelessness post-release | 5682 | 34,367 | 165.1 | 1 |  | 1 |  |
| **Any convicted criminal code violations** | | |  |  |  |  |  |
| Post-release homelessness | 110 | 889 | 123.7 | 3.0 | (2.5-3.7) | 2.4 | (2.0-3.0) |
| No sheltered homelessness post-release | 1649 | 34367 | 48.0 | 1 |  | 1 |  |
| **Any convicted traffic violations** |  |  |  |  |  |  |  |
| Post-release homelessness | 29 | 889 | 32.6 | 0.5 | (0.3-0.7) | 0.5 | (0.3-0.7) |
| No sheltered homelessness post-release | 2561 | 34,367 | 74.5 | 1 |  | 1 |  |
| **Any other conviction incl. weapon and illicit drug violations** |  |  |  |  |  |  |  |
| Post-release homelessness | 58 | 889 | 65.2 | 1.7 | (1.3-2.3) | 1.6 | (1.2-2.0) |
| No sheltered homelessness post-release | 1472 | 34,367 | 42.8 | 1 |  | 1 |  |
| IR=incidence rate, IRR=incidence rate ratio  ^*^Adjusted for sex, age, and calendar time. ^†^Further adjusted for country of origin, highest educational level, single status, length of imprisonment, and any psychiatric disorder including substance use disorders. | | | | | | | |
| **Supplementary table 13. Incidence rate ratio of recidivism within two years after prison release by post-release homelessness and type of the new conviction, 2001-2021** | | | | | | | |

|  |  |  |  |  |  | | |  |
| --- | --- | --- | --- | --- | --- | --- | --- | --- |
|  | **Cases** | **Person-years** | **IR/1000** | **IRR^*^** | **95% CI** | | |  |
| **Males** |  |  |  |  |  | | |  |
| **Total** | 5427 | 30793 | 176.2 |  |  | | |  |
| **Homeless shelter contact after release** |  |  |  |  |  | | |  |
| Yes | 168 | 743 | 226.0 | 1.4 | (1.2-1.7) | | |  |
| No | 5259 | 30,050 | 175.0 | 1 |  | | |  |
| **Homelessness after prison release in combination with psychiatric disorders** | | |  |  |  | | |  |
| **Mental illness** |  |  |  |  |  | | |  |
| Homelessness and mental illness | 77 | 285 | 270.2 | 1.8 | (1.4-2.2) | | |  |
| Homelessness and no mental illness | 91 | 458 | 198.5 | 1.3 | (1.0-1.6) | | |  |
| No homelessness and mental illness | 1044 | 5409 | 193.0 | 1.2 | (1.1-1.2) | | |  |
| None | 4215 | 24,641 | 171.1 | 1 |  | | |  |
| **Severe mental illness^†^** |  |  |  |  |  | | |  |
| Homelessness and severe mental illness | 21 | 77 | 271.6 | 1.9 | (1.3-3.0) | | |  |
| Homelessness and no severe mental illness | 147 | 666 | 220.7 | 1.4 | (1.2-1.6) | | |  |
| No homelessness and severe mental illness | 154 | 871 | 176.8 | 1.1 | (0.9-1.3) | | |  |
| None | 5105 | 29,179 | 175.0 | 1 |  | | |  |
| **Alcohol use disorder** |  |  |  |  |  | | |  |
| Homelessness and alcohol use disorder | 38 | 166 | 229.0 | 1.5 | (1.1-2.1) | | |  |
| Homelessness and no alcohol use disorder | 130 | 577 | 225.1 | 1.4 | (1.2-1.7) | | |  |
| No homelessness and alcohol use disorder | 583 | 3202 | 182.1 | 1.2 | (1.1-1.3) | | |  |
| None | 4676 | 26,848 | 174.2 | 1 |  | | |  |
| **Drug use disorder** |  |  |  |  |  | | |  |
| Homelessness and drug use disorder | 43 | 136 | 316.5 | 2.1 | (1.6-2.9) | | |  |
| Homelessness and no drug use disorder | 125 | 608 | 205.762 | 1.3 | (1.1-1.6) | | |  |
| No homelessness and drug use disorder | 433 | 1876 | 230.849 | 1.5 | (1.3-1.6) | | |  |
| None | 4826 | 28,174 | 171.291 | 1 |  | | |  |
| **Substance misuse comorbidity^†^** |  |  |  |  |  | | |  |
| Homelessness and substance misuse comorbidity | 48 | 141 | 341.014 | 2.2 | (1.7-3.0) | | |  |
| Homelessness and no substance misuse comorbidity | 120 | 603 | 199.141 | 1.3 | (1.1-1.5) | | |  |
| No homelessness and substance misuse comorbidity | 440 | 2174 | 202.437 | 1.3 | (1.2-1.4) | | |  |
| None | 4819 | 27,876 | 172.870 | 1 |  | | |  |
| **Females** |  |  |  |  |  | | |  |
| **Total** | 452 | 4463 | 101.3 |  |  | | |  |
| **Homeless shelter contact after release** |  |  |  |  |  | | |  |
| Yes | 29 | 146 | 198.6 | 2.2 | (1.5-3.2) | | |  |
| No | 423 | 4317 | 98.0 | 1 |  | | |  |
| **Homelessness after prison release in combination with psychiatric disorders** | | |  |  |  | | |  |
| **Mental illness** |  |  |  |  |  | | |  |
| Homelessness and mental illness | 19 | 83 | 229.6 | 2.9 | (1.8-4.7) | | |  |
| Homelessness and no mental illness | 10 | 63 | 158.0 | 2.0 | (1.0-3.7) | | |  |
| No homelessness and mental illness | 179 | 1467 | 122.0 | 1.4 | (1.2-1.7) | | |  |
| None | 244 | 2850 | 85.6 | 1 |  | | |  |
| **Severe mental illness^†^** |  |  |  |  |  | | |  |
| Homelessness and severe mental illness | … | … | … | … | … | | |  |
| Homelessness and no severe mental illness | … | … | … | … | … | | |  |
| No homelessness and severe mental illness | … | … | … | … | … | | |  |
| None | … | … | … | … | … | | |  |
| **Alcohol use disorder** |  |  |  |  |  | | |  |
| Homelessness and alcohol use disorder | 11 | 35 | 319.0 | 4.5 | (2.4-8.2) | | |  |
| Homelessness and no alcohol use disorder | 18 | 112 | 161.4 | 1.8 | (1.1-2.8) | | |  |
| No homelessness and alcohol use disorder | 70 | 605 | 115.7 | 1.3 | (1.0-1.6) | | |  |
| None | 353 | 3712 | 95.1 | 1 |  | | |  |
| **Drug use disorder** |  |  |  |  |  | | |  |
| Homelessness and drug use disorder | 11 | 40 | 277.8 | 3.3 | (1.8-6.0) | | |  |
| Homelessness and no drug use disorder | 18 | 106 | 169.1 | 1.9 | (1.2-3.1) | | |  |
| No homelessness and drug use disorder | 75 | 593 | 126.6 | 1.4 | (1.1-1.7) | | |  |
| None | 348 | 3724 | 93.4 | 1 |  | | |  |
| **Substance misuse comorbidity^†^** |  |  |  |  |  | | |  |
| Homelessness and substance misuse comorbidity | 10 | 50 | 199.3 | 2.5 | (1.4-4.8) | | |  |
| Homelessness and no substance misuse comorbidity | 19 | 96 | 198.3 | 2.2 | (1.4-3.5) | | |  |
| No homelessness and substance misuse comorbidity | 86 | 701 | 122.8 | 1.4 | (1.1-1.8) | | |  |
| None | 337 | 3617 | 93.2 | 1 |  | | |  |
| IR=incidence rate, IRR=incidence rate ratio  ^*^Adjusted for age and calendar time. **^†^**Severe mental illness defined as any schizophrenia spectrum disorder or any bipolar disorder; Substance misuse comorbidity defined as any mental illness and any alcohol or drug use disorder. | | | | | | |  |  |
| **Supplementary table 14. Incidence rate ratio of recidivism within two years after prison release by sex, 2001-2021** | | | | | | |  |  |
|  | **Cases** | **Person-years** | **IR/1000** | **IRR^*^** | | **95% CI** | | |
| **People serving a sentence** |  |  |  |  | |  | | |
| **Total** | 4770 | 29,656 | 160.8 |  | |  | | |
| **Homeless shelter contact after release** |  |  |  |  | |  | | |
| Yes | 164 | 760 | 215.8 | 1.5 | | (1.3-1.7) | | |
| No | 4606 | 28,896 | 159.4 | 1 | |  | | |
| **Homelessness after prison release in combination with psychiatric disorders** | | |  |  | |  | | |
| **Mental illness** |  |  |  |  | |  | | |
| Homelessness and mental illness | 74 | 275 | 268.7 | 2.0 | | (1.6-2.5) | | |
| Homelessness and no mental illness | 90 | 485 | 185.7 | 1.3 | | (1.0-1.6) | | |
| No homelessness and mental illness | 906 | 5372 | 168.6 | 1.2 | | (1.1-1.3) | | |
| None | 3700 | 23,524 | 157.3 | 1 | |  | | |
| **Severe mental illness^†^** |  |  |  |  | |  | | |
| Homelessness and severe mental illness | 13 | 44 | 297.4 | 2.3 | | (1.3-4.0) | | |
| Homelessness and no severe mental illness | 151 | 716 | 210.8 | 1.4 | | (1.2-1.7) | | |
| No homelessness and severe mental illness | 71 | 509 | 139.6 | 1.0 | | (0.8-1.2) | | |
| None | 4535 | 28,387 | 159.8 | 1 | |  | | |
| **Alcohol use disorder** |  |  |  |  | |  | | |
| Homelessness and alcohol use disorder | 40 | 171 | 233.6 | 1.7 | | (1.3-2.4) | | |
| Homelessness and no alcohol use disorder | 124 | 589 | 210.6 | 1.4 | | (1.2-1.7) | | |
| No homelessness and alcohol use disorder | 560 | 3340 | 167.7 | 1.2 | | (1.1-1.3) | | |
| None | 4046 | 25,557 | 158.3 | 1 | |  | | |
| **Drug use disorder** |  |  |  |  | |  | | |
| Homelessness and drug use disorder | 37 | 138 | 267.7 | 2.0 | | (1.5-2.8) | | |
| Homelessness and no drug use disorder | 127 | 622 | 204.2 | 1.4 | | (1.2-1.7) | | |
| No homelessness and drug use disorder | 384 | 2003 | 191.7 | 1.4 | | (1.2-1.5) | | |
| None | 4222 | 26,893 | 157.0 | 1 | |  | | |
| **Substance misuse comorbidity^†^** |  |  |  |  | |  | | |
| Homelessness and substance misuse comorbidity | 42 | 143 | 293.4 | 2.2 | | (1.7-3.0) | | |
| Homelessness and no substance misuse comorbidity | 122 | 617 | 197.7 | 1.4 | | (1.1-1.6) | | |
| No homelessness and substance misuse comorbidity | 385 | 2281 | 168.8 | 1.2 | | (1.1-1.4) | | |
| None | 4221 | 26,615 | 158.6 | 1 | |  | | |
| **People on remand** |  |  |  |  | |  | | |
| **Total** | 1109 | 5600 | 198.0 |  | |  | | |
| **Homeless shelter contact after release** |  |  |  |  | |  | | |
| Yes | 33 | 129 | 255.3 | 1.6 | | (1.1-2.3) | | |
| No | 1076 | 5471 | 196.7 | 1 | |  | | |
| **Homelessness after prison release in combination with psychiatric disorders** | | |  |  | |  | | |
| **Mental illness** |  |  |  |  | |  | | |
| Homelessness and mental illness | 22 | 92 | 238.6 | 1.8 | | (1.2-2.8) | | |
| Homelessness and no mental illness | 11 | 37 | 297.0 | 1.6 | | (0.9-2.9) | | |
| No homelessness and mental illness | 317 | 1505 | 210.7 | 1.2 | | (1.1-1.4) | | |
| None | 759 | 3966 | 191.4 | 1 | |  | | |
| **Severe mental illness^†^** |  |  |  |  | |  | | |
| Homelessness and severe mental illness | 12 | 55 | 218.6 | 1.7 | | (0.9-2.9) | | |
| Homelessness and no severe mental illness | 21 | 74 | 282.4 | 1.6 | | (1.1-2.5) | | |
| No homelessness and severe mental illness | 108 | 575 | 187.7 | 1.2 | | (1.0-1.5) | | |
| None | 968 | 4896 | 197.7 | 1 | |  | | |
| **Alcohol use disorder** |  |  |  |  | |  | | |
| Homelessness and alcohol use disorder | 9 | 29 | 308.5 | 2.3 | | (1.2-4.5) | | |
| Homelessness and no alcohol use disorder | 24 | 100 | 239.8 | 1.5 | | (1.0-2.2) | | |
| No homelessness and alcohol use disorder | 93 | 468 | 198.9 | 1.2 | | (1.0-1.5) | | |
| None | 983 | 5003 | 196.5 | 1 | |  | | |
| **Drug use disorder** |  |  |  |  | |  | | |
| Homelessness and drug use disorder | 17 | 37 | 456.9 | 3.2 | | (2.0-5.2) | | |
| Homelessness and no drug use disorder | 16 | 92 | 173.8 | 1.1 | | (0.7-1.9) | | |
| No homelessness and drug use disorder | 124 | 465 | 266.7 | 1.8 | | (1.5-2.1) | | |
| None | 952 | 5006 | 190.2 | 1 | |  | | |
| **Substance misuse comorbidity^†^** |  |  |  |  | |  | | |
| Homelessness and substance misuse comorbidity | 16 | 48 | 334.7 | 2.5 | | (1.5-4.0) | | |
| Homelessness and no substance misuse comorbidity | 17 | 82 | 208.7 | 1.3 | | (0.8-2.1) | | |
| No homelessness and substance misuse comorbidity | 141 | 593 | 237.8 | 1.6 | | (1.3-1.9) | | |
| None | 935 | 4878 | 191.7 | 1 | |  | | |
|  |  |  |  |  | |  | | |
| IR=incidence rate, IRR=incidence rate ratio  ^*^Adjusted for sex, age, and calendar time. ^†^Severe mental illness defined as any schizophrenia spectrum disorder or any bipolar disorder; Substance misuse comorbidity defined as any mental illness and any alcohol or drug use disorder. | | | | | | | | |
| **Supplementary table 15. Incidence rate ratio of recidivism within two years after prison release by type of index-imprisonment, 2001-2021** | | | | | | | | |

1. Statistics Denmark. Documentation of statistics for Shelters 2021. 2021. <https://www.dst.dk/en/Statistik/dokumentation/documentationofstatistics/shelters> (accessed November 11, 2022).

2. Statistics Denmark. Imprisonments 2021. <https://www.dst.dk/en/Statistik/dokumentation/documentationofstatistics/imprisonments> (accessed November 20, 2021).

3. Schmidt M, Schmidt SA, Sandegaard JL, Ehrenstein V, Pedersen L, Sørensen HT. The Danish National Patient Registry: a review of content, data quality, and research potential. *Clin Epidemiol* 2015; **7**: 449-90.

4. Mors O, Perto GP, Mortensen PB. The Danish Psychiatric Central Research Register. *Scand J Public Health* 2011; **39**: 54-7.

5. National Board of Health. The Registry of Drug Abusers Undergoing Treatment. 2020. <https://sundhedsdatastyrelsen.dk/da/registre-og-services/om-de-nationale-sundhedsregistre/sygdomme-laegemidler-og-behandlinger/stofmisbrugere-i-behandling> (accessed March 5, 2021).

6. Schmidt M, Pedersen L, Sorensen HT. The Danish Civil Registration System as a tool in epidemiology. *Eur J Epidemiol* 2014; **29**: 541-9.

7. Statistics Denmark. Countries in Social Statistics [Lande i Personstatistik, v1:2021]. 2021. <https://www.dst.dk/da/Statistik/dokumentation/nomenklaturer/lande-psd> (accessed July 7, 2023).

8. Den Integrerede Database for Arbejdsmarkedsforskning (IDA). *Center for Registerforskning*, 2010. <http://ncrr.au.dk/registre/den-integrerede-database-for-arbejdsmarkedsforskning-ida/> (accessed 1/27/2012).
